# EEG-based high-performance depression state recognition

**DOI:** 10.3389/fnins.2023.1301214

**Published:** 2024-01-31

**Authors:** Zhuozheng Wang, Chenyang Hu, Wei Liu, Xiaofan Zhou, Xixi Zhao

**Affiliations:** ^1^Faculty of Information Technology, Beijing University of Technology, Beijing, China; ^2^The National Clinical Research Center for Mental Disorders & Beijing Key Laboratory of Mental Disorders, Beijing Anding Hospital, Capital Medical University, Beijing, China

**Keywords:** scalp EEG signals, feature dimension reduction, feature-weighted fusion, graph convolutional neural network, recognition of depressive state

## Abstract

Depression is a global disease that is harmful to people. Traditional identification methods based on various scales are not objective and accurate enough. Electroencephalogram (EEG) contains abundant physiological information, which makes it a new research direction to identify depression state. However, most EEG-based algorithms only extract the original EEG features and ignore the complex spatiotemporal information interactions, which will reduce performance. Thus, a more accurate and objective method for depression identification is urgently needed. In this work, we propose a novel depression identification model: W-GCN-GRU. In our proposed method, we censored six sensitive features based on Spearman’s rank correlation coefficient and assigned different weight coefficients to each sensitive feature by AUC for the weighted fusion of sensitive features. In particular, we use the GCN and GRU cascade networks based on weighted sensitive features as depression recognition models. For the GCN, we creatively took the brain function network based on the correlation coefficient matrix as the adjacency matrix input and the weighted fused sensitive features were used as the node feature matrix input. Our proposed model performed well on our self-collected dataset and the MODMA datasets with a accuracy of 94.72%, outperforming other methods. Our findings showed that feature dimensionality reduction, weighted fusion, and EEG spatial information all had great effects on depression recognition.

## 1 Introduction

In contemporary society, people face more and more pressure from life and work, and various mental diseases appear one after another. Depression is currently the most common and high-incidence mental disorder, fundamentally affecting people’s normal lives (World Federation for Mental Health, 2012). Symptoms exhibited by depression are usually low mood lasting for more than two weeks, increasingly slow thinking, lower and lower self-appraisal, impairment of cognitive function, etc. Patients with major depressive disorder even show self-harming and life-threatening behaviours. More than 350 million people worldwide suffer from varying degrees of depressive states. By 2030, depression is expected to be the number one burden disease in the world. The number of pan-depressed people in China is currently about 95 million, and about 16% of people will suffer depression at some point in their lives (World Health Organization, 2017). The global crisis of depression is still very serious, and concerted efforts are needed from all sectors of society to arouse a wider concern about depression.

The term “depression” is a more rigorous term. A medically accurate diagnosis is required for a person to be diagnosed with depression. While most people may just have a tendency to be depressed or suffer from depressed mood for a short period of time, it is a short-term disorder of the mind that can be called a depressive state. Rates of consultation and diagnosis are low, both for people with diagnosed depression and for those in a depressed state. The first reason is that the symptoms of people in a state of depression are not obvious and last for a short period of time, so it is difficult to draw attention to them; the second reason is that in our country, there are a large number of people with mental illnesses, but there is not yet a sound medical system and professional psychiatrists. Recognition of depressive states goes through different stages of development. Traditional depression identification is based on a variety of scales that doctors use to give diagnosis and treatment results. This has the problem of strong objectivity and low accuracy. With the development of brain–computer interface (BCI) and deep learning, depression recognition by electroencephalogram (EEG) has become popular. However, the current EEG-based depression recognition has two limitations. Firstly, researchers have not processed the features too much and send the original features directly into the classifier, which prevents obtaining the optimal feature information; secondly, researchers have overlooked the topological relationship between the electrodes, neglecting the complex spatiotemporal information among brain regions. All these problems can degrade the performance of depression state recognition. Therefore, how to find an objective and efficient method to identify depressive states is a challenge for current research.

EEG, known for its obvious advantages such as high temporal resolution, high sensitivity, relatively low cost, easy operation, and non-invasiveness, is now effective and frequently used for depressive state recognition. Many new papers have demonstrated that using linear characteristics of EEG to identify depressed patients is a feasible approach. Based on the EEG data of 34 depressed patients and 30 healthy people, Mahato and Paul (2019) achieved the highest accuracy of 91.67% with the classifier by using the linear features of band power and interhemispheric asymmetry. Zhaoyan and Xiaoyan (2022) used support vector machines to identify depression binary classification based on resting state EEG signal, achieving the best classification results of 94.24% accuracy, 92.35% recall rate, and 96.23% accuracy. In 2019, Ay et al. (2019) proposed a deep mixing model that used Convolutional Neural Network (CNN) and Long Short Term Memory (LSTM) structure to detect EEG signals of depression, and the classification accuracy of EEG in both left and right hemispheres reached more than 85%. In 2022, Liu et al. (2022) explored an end-to-end depression recognition method based on EEGnet, and got 90.98% accuracy rate by directly inputting EEG into neural network for recognition of patients with major depressive disorder. In addition, EEG also has the characteristics of nonlinear, irregular, and high complexity, so the feature extraction of EEG based on nonlinear dynamics is a further study on depression recognition. Shuting Sun et al. (2020) used L, NL, PLI, and NM features to achieve 75.8% accuracy in a sample of 24 severely depressed individuals and 29 normal subjects. Hosseinifard et al. (2013) used a combination of linear and nonlinear methods to identify depression, and the results showed that the nonlinear analysis method would achieve higher accuracy. Similarly, Akar et al. (2015) adopted a nonlinear method for depression binary recognition and concluded that the brain complexity of people with depression is higher. Puthankattil and Joseph (2014) used signal entropy as a feature of their study, and the experimental results showed that depressed patients have lower ApEn values than normal people and depression causes a decrease in both the complexity and predictability of EEG signals. Faust et al. (2014) found that depressed patients have lower sample entropy and approximate entropy values than healthy people, suggesting that depressed patients have reduced EEG complexity and increased predictability. Hajian et al. (2019) obtained 90% accuracy based on fuzzy entropy, KFD, and HFD characteristics in a sample of 60 subjects with varying degrees of depression. In particular, Zhang Lu (2022) studied feature selection algorithms in 2022. Based on different feature selection algorithms and oversampling techniques, the accuracy of 82.16% is obtained by taking the oversampling feature matrix as the network input. Currently, the latest research focuses on depression recognition in brain functional network and generates functional connection matrix through different coupling methods. In 2020, Rong (2020) constructed a convolutional neural network recognition model for mild depression based on a functional connection matrix and obtained a recognition rate of 80.74%. In 2020, Chen (2020) found that the differences of brain functional networks examined by different functional connectivity approaches were different, and the final results showed that coherent brain functional networks combined with SVM had the best dichotomous recognition results with 90% accuracy. In 2021, Chang et al. (2022) obtained 87.67% accuracy for EEG-based Parkinson’s disease recognition based on sparse graph convolutional neural network with attention. In 2022, Zhu et al. (2022) introduced a learning weight matrix into the input layer of the graph convolutional neural (GCN) network to optimize the brain functional network, achieving a recognition accuracy rate of 96.50% among normal and depressed people. In particular, Chang et al. (2023) obtained 87.67% accuracy for EEG-based Parkinson’s disease recognition based on attention-based sparse graph convolutional neural network.

However, for the feature layer, no matter whether using linear features, nonlinear features, or the combination of two types of features, the original features are only sent into the neural network without further optimization processing, which is easy to produce redundant features and affect the recognition performance. In addition, for the construction of the adjacency matrix of the brain functional network, most studies define the connection relationship by the spatial distance between nodes, and direct connection exists only when the spatial distance is very close. However, there are some long-distance connections in the brain. Simply defining a connection in terms of distance can lead to the loss of important information. In response to the above problem, we proposed our model W-GCN-GRU based on weighted sensitive features for depression identification. In this model, the original features are firstly reduced and weighted, and then the weighted fused features are fed into the network cascaded by GCN and GRUs. The cascaded network extracts not only EEG temporal information but also EEG spatial information. In particular, Spearman’s rank correlation coefficient was introduced to construct an adjacency matrix to avoid the loss of important spatial information.

## 2 Materials and methods

### 2.1 Data sources

In this study, the data sources include open-source MODMA datasets and self-collected datasets. Each dataset is resting state and both include healthy people and depressed people.

#### 2.1.1 MODMA dataset

The MODMA dataset is a multimodal dataset released by Lanzhou University for the analysis of mental disorders. For EEG data, the experiment selected a 128-channel HydroCel geodesic sensor network and Net Station acquisition software to record continuous EEG signals. Participants were asked to remain awake and still, without unnecessary eye movements and blinking. One hundred and twenty-eight resting state EEG data were obtained from the experiment. The specific clinical information is shown in Table 1. For the sake of time performance and computational efficiency, this study selected data from 16 channels (Fp1/2, F3/4, C3/4, P3/4, O1/2, F7/8, T3/4, and T5/6). Previous studies have also demonstrated the feasibility of using these electrodes for depression state recognition (Zhang and He, 2018).

**Table 1:** Clinical information for MODMA.

	Depressed patients	Healthy control subjects
Number of people	24	29
Gender (male/female)	13/11	20/9
Age (years)	16–56	18–55
Sampling frequency	250 Hz	
Reference electrode	Cz	
Single acquisition time	5 minutes	

#### 2.1.2 Self-collecting dataset

**Figure 1:**
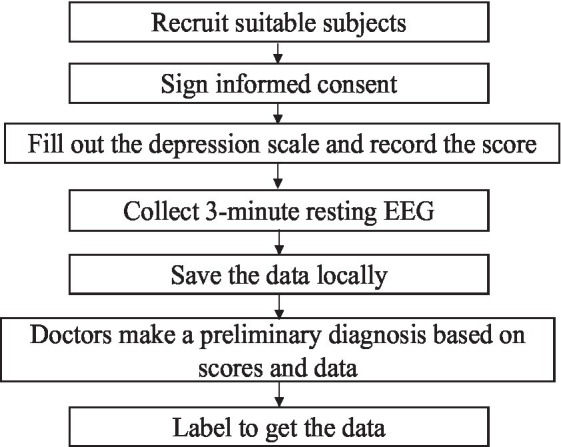
Process of self-collecting data.

An 18-channel dry electrode cap was used to collect resting EEG data in this study. Sixteen active electrodes (Fp1/2, F3/4, C3/4, P3/4, O1/2, F7/8, T3/4, and T5/6) could collect EEG data of 16 channels. The electrode cap parameters are as follows: (1) DC offset voltage: ≤ 180uV; (2) potential drift: ≤ 25 uV/h; (3) AC impedance: ≤ 0.15KΩcm^2^; and (4) resistance: ≤ 5 Ω. The specific signal acquisition process is shown in Figure 1. The basic metrics of the acquisition device during the acquisition process are shown in Table 2.

The experimental paradigm for EEG signal acquisition needs to be designed before data collection. The subjects were from the Mental Health Centre of Beijing Institute of Technology, and all acquisition experiments in this study were ethically certified. Before the experiment, all subjects signed an informed consent form. During the experiment, the subjects were first asked to fill out the Self-Depression Scale (SDS) and record the scores, and then they were put in a quiet room, sat in a chair, closed their eyes, relaxed and did not move, and a single 3-minute resting-state EEG signal was collected and stored locally. The study was based on the scale scores as well as the initial diagnosis made by the doctors at the partner hospital and labelled the data.

**Table 2:** Collects device indicators

**Basic indicators**	
Channel number	16
Sampling frequency	250Hz
Operating voltage	6V
Bias current (electronics)	200pA
Baud rate	115200
Resolution	0.01μV, 24bit
Impedance	<50KΩ
Single collection duration	3 minutes

### 2.2 Data preprocessing

EEG signals are very weak, easy to be interfered, and often doped with a variety of endogenous and exogenous artifacts. The exogenous artifact is mainly power frequency interference. Endogenous artifacts are mainly electrocardiogram (ECG), electromyography (EMG), and electrooculography (EOG) interferences that overlap with EEG in the frequency band (Walczak and Chokroverty, 1994). Therefore, preprocessing is necessary to obtain a relatively pure EEG.

**Figure 2:**
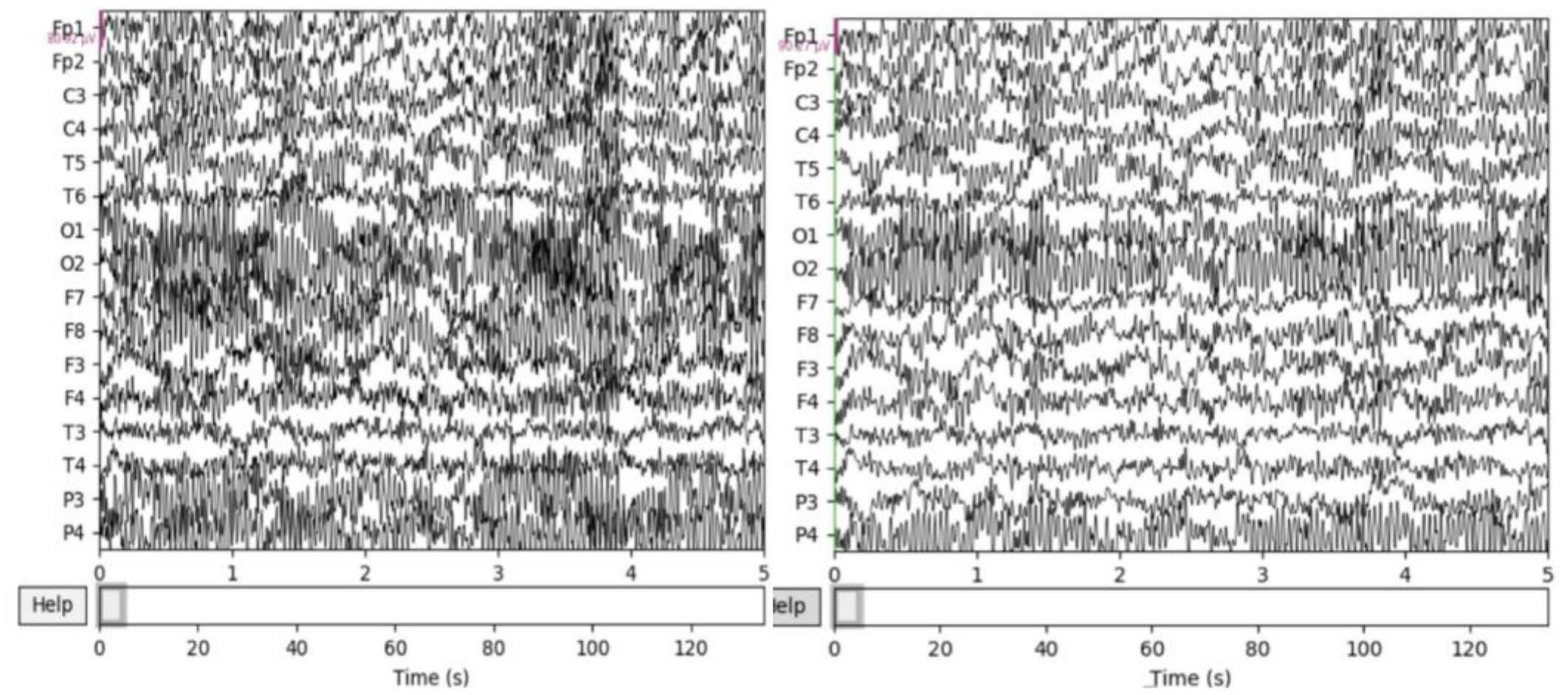
ICA before and after artifact elimination comparison.

The resting EEG was further processed with the MATLAB EEGLAB toolbox and several plugins. First, a 50 Hz notch filter is used to remove severe power frequency interference. Second, a band pass filter of 0.5–50 Hz was used to remove some endogenous artifacts. Third, use baseline correction to remove baseline differences between data segments caused by low frequency drift or other artifacts. Specifically, the average baseline value is removed from each time period to eliminate any bias. Fourth, there are still many endogenous artifacts overlapping with EEG in the frequency band, which are removed by independent principal component analysis (ICA) in this paper. Figure 2 compares the signals before and after processing, and it can be seen that the preprocessed signals are obviously more pure. Finally, EEG was cut every second to increase the sample size and make the experiment more convincing.

### 2.3 Feature extraction and processing

#### 2.3.1 Feature extraction

After reading a large number of literatures, in order to obtain more abundant EEG information, this study extracted 23-dimensional primitive EEG features: 7 time domain features, 10 frequency domain features, and 6 nonlinear features. Time domain features are Max, Min, Mean, Var, ppMean, Kurtosis, and Skewness. Frequency domain features are E (δ), E (θ), E (α), E (β), $E_{\mathrm{all}}$ $E\;\left(\delta \right)+E\;\left(\theta \right)+E\;\left(\alpha \right)+E\;\left(\beta \right)$ $E_{\delta \_\mathrm{rela}}$ $E\;\left(\delta \right)/E_{\mathrm{all}}$ $E_{\theta \_\mathrm{rela}}$ $E\;\left(\theta \right)/E_{\mathrm{all}}$ $E_{\alpha \_\mathrm{rela}}\;$ $E\;\left(\alpha \right)/E_{\mathrm{all}}$ $E_{\beta \_\mathrm{rela}}$ $E\;\left(\beta \right)/E_{\mathrm{all}}$ and PSD. Nonlinear features are HFD (Higuchi fractal dimension), Hjorth parameter (activity, mobility, and complexity), SampEn, and DFAs (detrended fluctuation analysis).

#### 2.3.2 Feature dimension reduction

Although the original feature contains more comprehensive information, with the deepening of the calculation, the model parameters will increase significantly and there will be redundant features, which will greatly increase the computational complexity of the network. Therefore, the feature dimension must be reduced.

Common correlation coefficients include Pearson’s correlation coefficient (PCC) (Xia et al., 2017) and Spearman’s rank correlation coefficient (SCC) (Stephanou and Varughses, 2021). Spearman’s rank correlation coefficient is more robust to abnormal data and measures trend correlation rather than nonlinear correlation among variables. In summary, it is more applicable than Pearson’s correlation coefficient (Yuan et al., 2023). Therefore, Spearman’s correlation coefficient is introduced in this paper to measure the correlation among features. The equation (1) is shown as follows.


(1) $$\rho \left(X,Y\right)=\frac{\sum_{i=1}^{n}\left(r_{x_{i}}-\bar{r_{x}}\right)\left(r_{y_{i}}-\bar{r_{y}}\right)}{\sqrt{\sum_{i=1}^{n}{\left(r_{x_{i}}-\bar{r_{x}}\right)}^{2}{\left(r_{y_{i}}-\bar{r_{y}}\right)}^{2}}}$$

The Spearman’s correlation coefficient is between −1 and 1, where −1 represents complete inverse correlation, 0 represents no correlation, and 1 represents complete positive correlation. Generally, the absolute values of coefficient and correlation are shown in Table 3.

**Table 3:** Spearman’s correlation.

|SCC|	Correlation
(0–0.2]	Extremely weak or irrelevant
(0.2–0.4]	Weak
(0.4–0.6]	Moderate strength
(0.6–0.8]	Strong
(0.8–1.0]	Extremely strong

**Figure 3:**
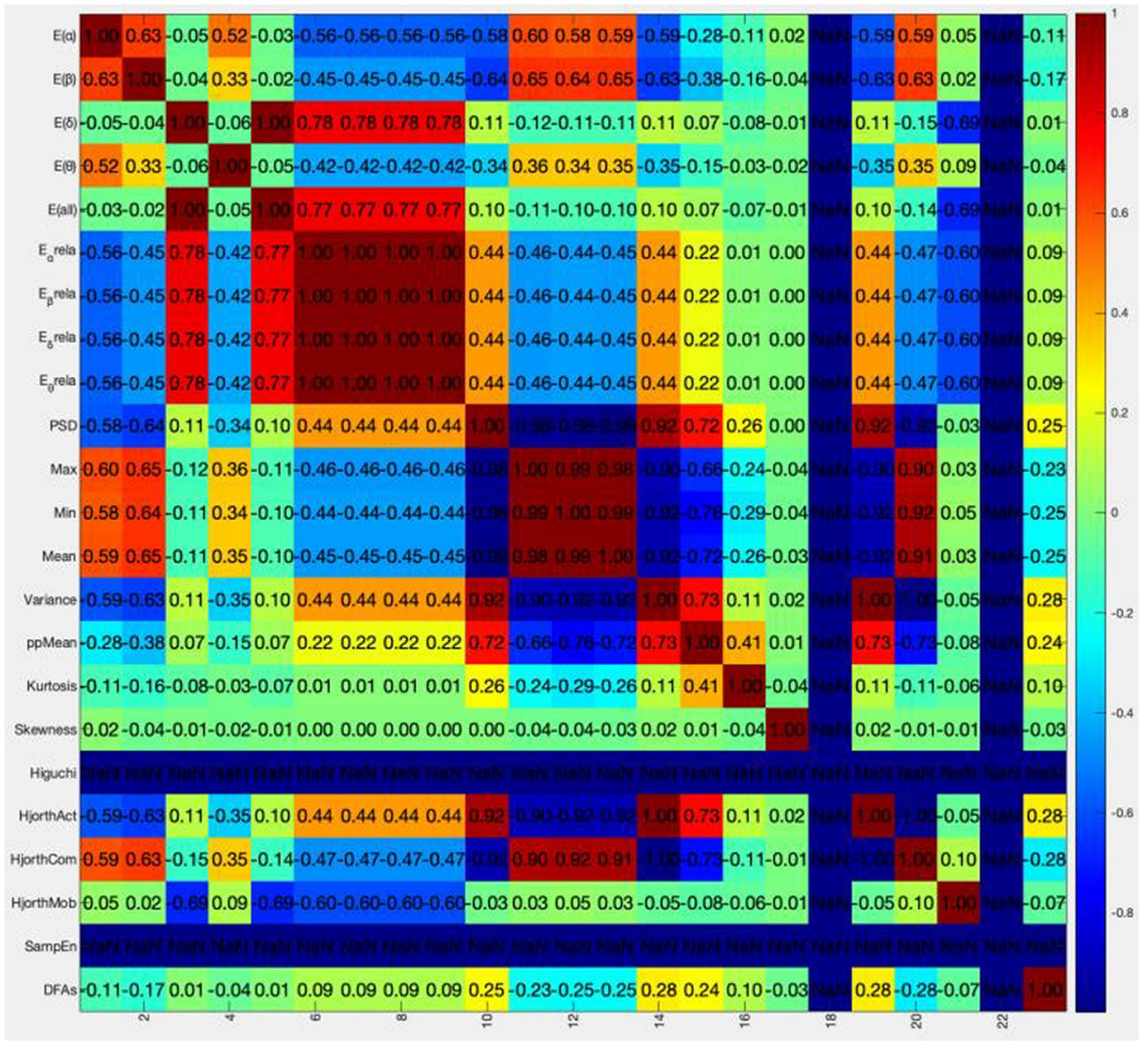
Original feature correlation coefficient color diagram.

The colormap is drawn using colormap function and jet parameter in MATLAB. The correlation coefficient matrix is visualized between 23 dimensional original features. The results are shown in Figure 3.

The abscissa in the above figure is the number of feature values, and the ordinate is the name of the feature values. It is obvious that the correlation coefficients between Higuchi and SampEn features and other features are all NaN (not a number), which is caused by the zero corresponding standard deviation in the calculation process. These two features will not be considered in the following analysis. In order to directly know the number of different correlations between each feature and other features, Table 4 is obtained.

**Table 4:** Correlation statistics.

Feature name	(0.0–0.2]	(0.2–0.4]	(0.4–0.6]	(0.6–0.8]	(0.8–1.0]	the ratio between 0 and 0.4
E(α)	6	1	12	1	1	33%
E(β)	6	2	4	8	1	38%
E(δ)	14	0	0	5	2	**67%**
E(θ)	7	8	5	0	1	71%
$E_{all}$	14	0	0	5	2	**67%**
$E_{\mathrm{\alpha relative}}$	3	1	11	2	4	19%
$E_{\mathrm{\beta relative}}$	3	1	11	2	4	19%
$E_{\mathrm{\delta relative}}$	3	1	11	2	4	19%
$E_{\mathrm{\theta relative}}$	3	1	11	2	4	19%
PSD	4	3	5	2	7	33%
max_	4	3	5	2	7	33%
min_	4	3	5	2	7	33%
means	4	3	5	2	7	33%
var	5	2	5	2	7	33%
ppMean	5	7	1	7	1	57%
**Kurtosis**	15	4	1	0	1	**90%**
**Skewness**	20	0	0	0	1	**95%**
Hjorth activity	5	2	5	2	7	33%
Hjorth complexity	5	2	5	2	7	33%
**Hjorth mobility**	14	0	4	2	1	**67%**
**DFAs**	12	8	0	0	1	**95%**

The bold values is the correlation value (Quantitative indicators).

**Figure 4:**
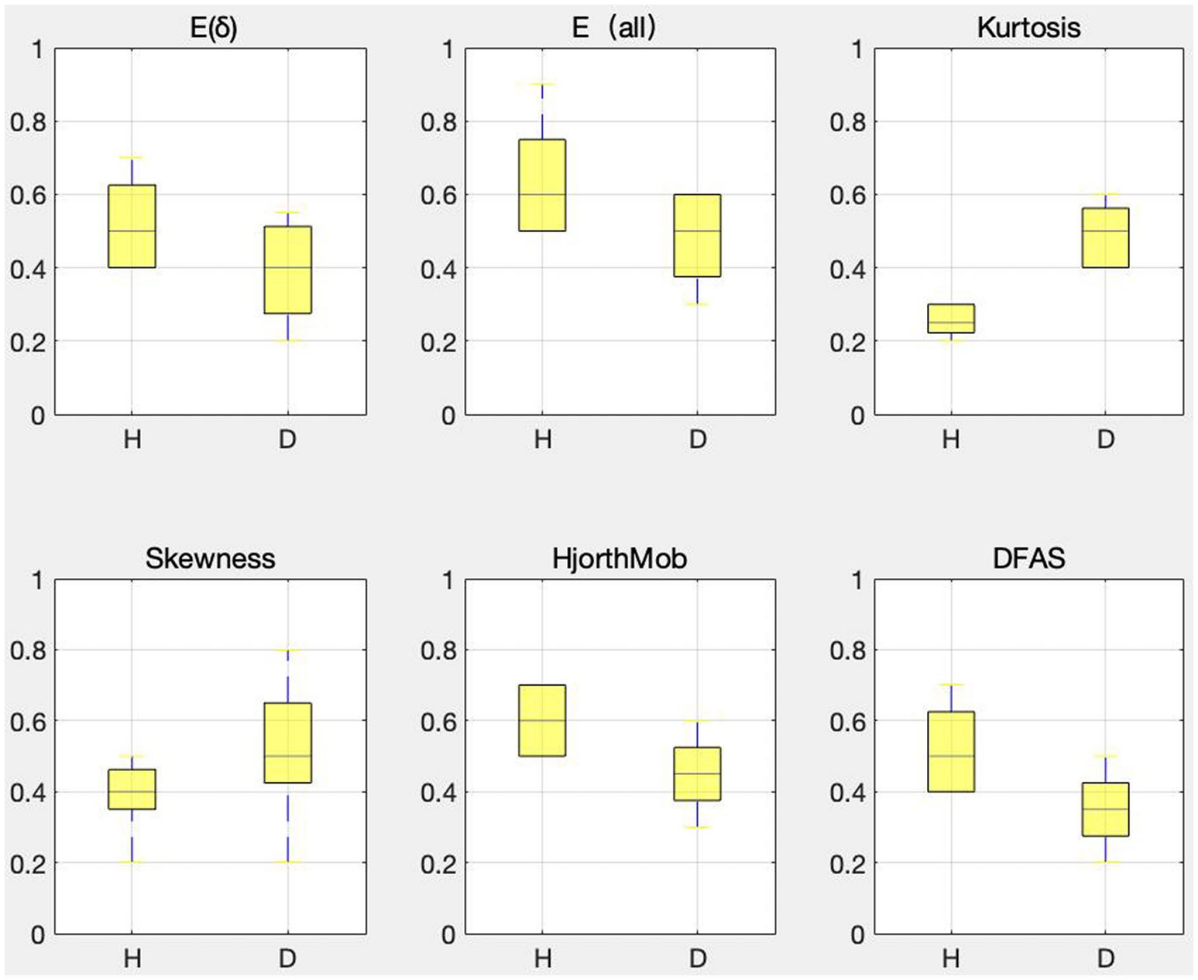
Differences in the distribution of sensitive features.

The last column in Table 4 shows the percentage of correlation between each feature and other features in the range of 0–0.4, and the selection ratio is greater than or equal to 67%. Therefore, E (δ), $E_{\mathrm{all}}$ , Kurtosis, Skewness, Hjorth mobility, and DFAs are the six features after feature dimension reduction, which are called sensitive features in this paper. To verify the validity of the sensitive feature, a differential distribution experiment was conducted. Before the experiment, mapminmax function of MATLAB was used to normalize the eigenvalues to [−1,1]. Figure 4 shows the distribution difference between the 6 sensitive features between normal and depression, with H representing normal and D representing depression. The results show that the sensitivity of six sensitive features to depression identification and their selection as sensitive features in this study is feasible.

#### 2.3.3 Weighted fusion of sensitive features

Six sensitive features have been able to fully represent the EEG information. Nonetheless, the contribution of different sensitive features to depression identification is not necessarily the same. Therefore, in order to improve the high performance of depression identification, this study assigns different weights to each sensitive feature. Inspired by Peng et al. (2023), the weight coefficient of sensitive features in this paper requires the use of parameter AUC (area under the curve), that is, the area under receiver operating characteristic curve (ROC). AUC is often used as a measure of model evaluation. The horizontal axis of the ROC curve is the false positive rate FPR and the vertical axis is the true rate TPR. The FPR calculation is shown in equation (2)


(2) $$FPR=\frac{\left|FP\right|}{\left|FP\right|+\left|TN\right|}$$

**Figure 5:**
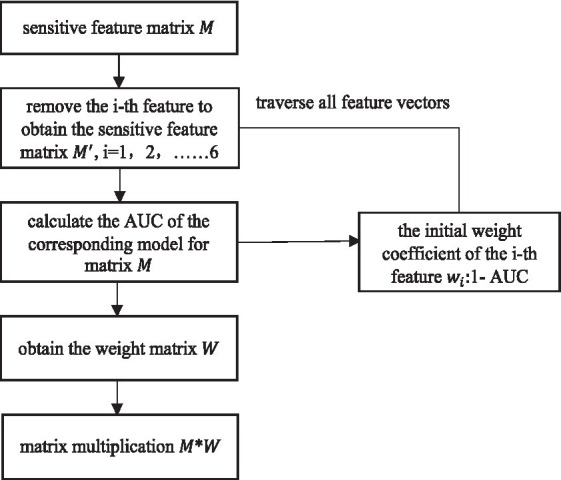
Weighted fusion flowchart.

The TPR is calculated using the same formula as the recall rate, i.e., equation (14). According to the meaning of the ROC curve, it can be seen that the AUC takes the value of [0,1].The closer the AUC value is to 1, the better the separability measure of the model is; when the AUC value is equal to 0.5, the model function is the same as the random guessing, just like flipping a coin, and at this time the model loses its predictive value; the closer the AUC value is to 0, the worse the separability measure of the model is. Six sensitive feature vectors form the sensitive feature matrix M. Starting from the first feature vector, M is traversed. First, obtain the feature matrix $M^{\prime }$ without the first feature vector, and then calculate the classification accuracy AUC of the model corresponding to $M^{\prime }$ . The classification effect of the sensitive feature matrix M is regarded as 1, 1-AUC as the weight of the first feature. After traversing all the features, a set of weight values is obtained and stored in the weight matrix W. Multiply the sensitive feature matrix M and weight matrix W for weighted fusion of feature layers. The weighted fusion flowchart is shown in Figure 5.

**Figure 6:**
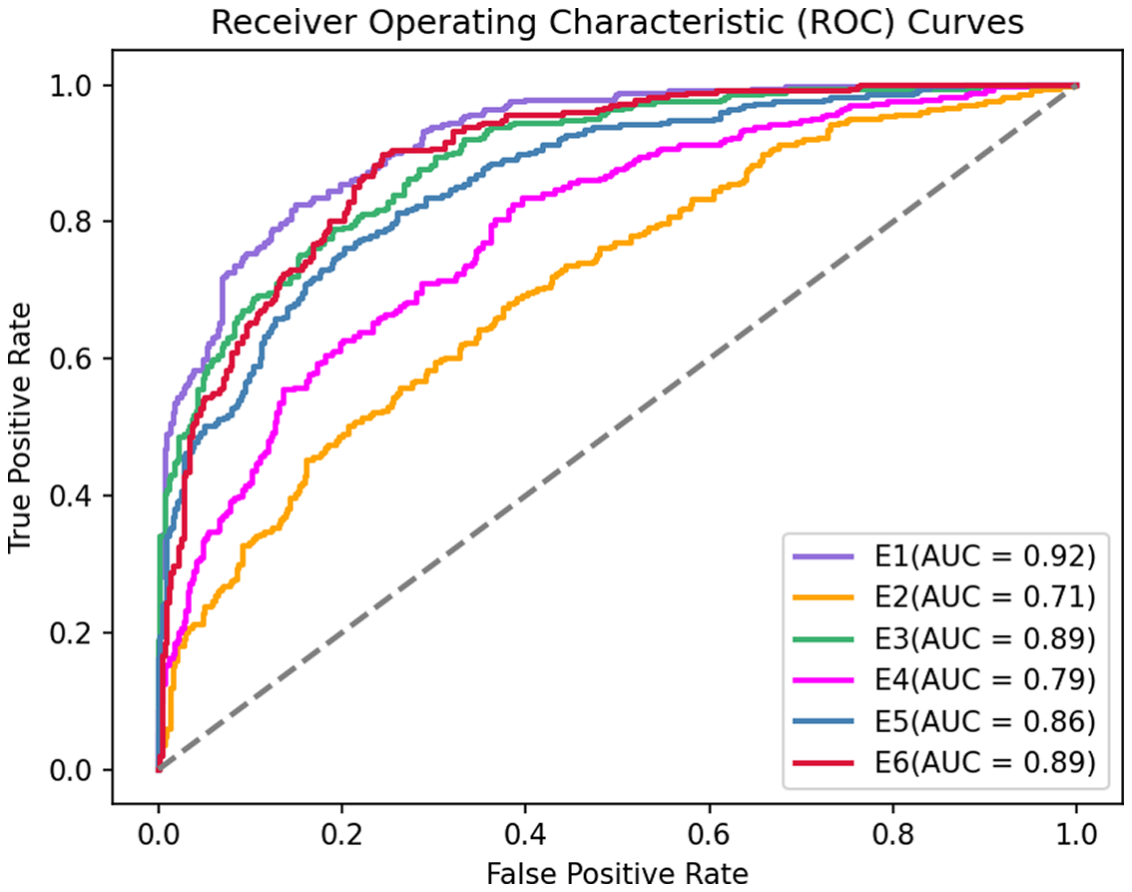
Sensitive feature weighted AUC curve.

For AUC, GRU network is used in this paper. The six ROC curves obtained from the six experiments are shown in Figure 6.

To meet the sum of six weight coefficients of 1, the initial weight coefficient is normalized. Table 5 presents the experimental results in detail. Finally, this study obtained the weighted fused feature as input to the model:0.08* $E\;\left(\delta \right)$ + 0.31* $E_{\mathrm{all}}$ +0.12* Kurtosis + 0.22* Skewness + 0.15* Hjorth mobility + 0.12* DFAS.

**Table 5:** Weight coefficient analysis.

Experiment	Sensitive features	AUC	Initial weight	Final weight
$E_{1}$	E(δ)	0.92	0.08	0.08
$E_{2}$	$E_{\mathrm{all}}$	0.71	0.29	0.31
$E_{3}$	Kurtosis	0.89	0.11	0.12
$E_{4}$	Skewness	0.79	0.21	0.22
$E_{5}$	Hjorth mobility	0.86	0.14	0.15
$E_{6}$	DFAS	0.89	0.11	0.12

### 2.4 The architecture of our model

EEG is a time series and the data have a backward and forward dependence in time. In deep learning algorithms, GRU is able to fully extract the temporal features of the data based on solving the gradient explosion and gradient disappearance caused by long time sequences. In addition, EEG signals are collected on channels with spatial position relationship. GCN can take node features and brain network structure information as input at the same time, considering all its neighbors and its own contained feature information, fully extracting the spatial features between data. Hence, in order to extract features with spatial and temporal correlation from a given EEG signal, this chapter proposes a hybrid neural network W-GCN-GRU depressive state recognition method based on weighted sensitive features to achieve high-performance recognition and classification of depressive states.

#### 2.4.1 GRU network structure

**Figure 7:**
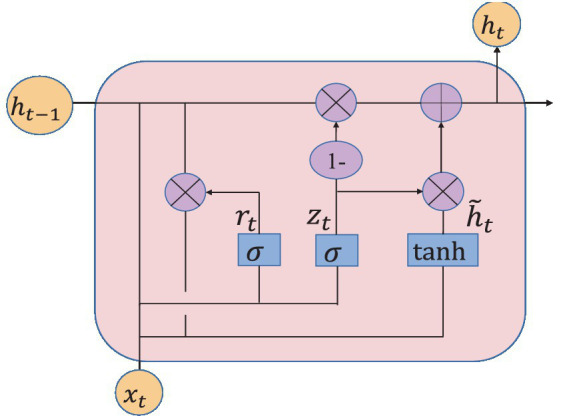
GRU network structure.

Gate Recurrent Unit (GRU) (Chung et al., 2014) is a type of Recurrent Neural Network (RNN) (Schmidhuber, 2015). Like long short-term memory (LSTM) (Hochreiter and Schmidhuber, 1997), it is also proposed to solve the problem of gradient explosion and gradient disappearance. But GRU has a simpler structure and better training effect compared to LSTM networks. In this study, a GRU layer is added to reflect the temporal correlation of EEG. The GRU’s special “gate” structure can delete or add information to the neuron state, allowing information to pass through selectively. The gate consists of a sigmoid neural network layer and a point-by-point multiplicator. Sigmoid outputs a number between 0 and 1 that determines how much information each neuron can transmit (Olimov et al., 2021). 0 indicates that all information cannot pass, and 1 indicates that all information can pass. The improvement of GRU is that it only has two door structures. One is the update gate $z_{t}$ , which is used to control the update of hidden unit status. The update gate determines how much information was retained in the current time step from the previous moment. Another is the reset gate $r_{t}$ , which defines how to combine the current input information with the previously saved memory. The GRU network is shown in Figure 7.

The great advantage of these two gates is that they can retain information in long-term sequences and do not delete it because it is not relevant to the prediction task.

The output of the update gate is shown in equation (3):


(3) $$z_{t}=\sigma \left(W_{z}x_{t}+W_{z}h_{t-1}+b_{z}\right)$$

The output of the reset door is calculated in equation (4):


(4) $$r_{t}=\sigma \left(W_{r}x_{t}+h_{t-1}+b_{r}\right)$$

The new memory contents are defined as equation (5):


(5) $$\tilde{h_{t}}=\tanh\;\left(W_{x}x_{t}+W_{h}\left(h_{t-1}*r_{t}\right)+b\right)$$

The memory content of the current time is defined as equation (6):


(6) $$h_{t}=\left(1-Z_{t}\right)*h_{t-1}+Z_{t}*\tilde{h_{t}},t=1,2,\ldots \ldots T$$

where $W_{Z}$ , $W_{r}$ , $W_{x}$ , and $W_{h}$ represent weight matrix; $b_{z}$ , $b_{r}$ , and b represent bias vector; subscripts z, r and h represent update gate, reset gate and hidden unit, respectively; σ represents sigmoid activation function; x(t) is the input vector at the t time step; h (t-1) is the output of the previous neuron.

#### 2.4.2 GCN network structure

The channels that collect EEG are distributed in different spatial positions, and the state of each channel and the relationship between channels are important factors for depression recognition. Such a relationship is analogous to an irregular graph structure, also called a topology. In graph data, there are not only nodal features (data of nodes) but also graph structure (how nodes are connected to each other). In addition, the structure around each node may be unique. Such flexible and complex data structure makes traditional convolutional neural networks no longer have their original advantages, so consider moving convolution operations from dealing with traditional Euclidean structured data to topologically structured graph data. Graph convolutional network (GCN) is a convolutional neural network that acts directly on the graph and uses its structural information for feature extraction. Like convolutional neural networks, it is usually composed of a convolutional layer, a pooling layer, an activation function, a fully connected layer, and so on.

There is a graph with N nodes. The input feature dimension of each node is D, and the features of all nodes will form an N × D feature matrix H; at the same time, an N × N Adjacency Matrix (A) is formed by analyzing the functional connection relationships among nodes. The inputs to the GCN model are the feature matrix H and the adjacency matrix A. The mode of propagation between layers of GCN is shown in equation (7).


(7) $$H^{\left(l+1\right)}=\sigma \left({\tilde{D}}^{-\frac{1}{2}}\tilde{A}{\tilde{D}}^{-\frac{1}{2}}H^{\left(l\right)}W^{\left(l\right)}\right)$$

σ is a nonlinear activation function, such as Sigmoid, ReLU, and Softmax. $\tilde{D}$ is the degree matrix of $\tilde{A}$ . $\tilde{A\;}$ *=* A + I, where I is the identity matrix. A is an adjacency matrix of one of the inputs to the model, and W is the weight matrix to be trained.

#### 2.4.3 Construction of adjacency matrix and feature matrix

For the feature matrix H, this study uses a weighted fusion of six sensitive features as the feature matrix for each node. The EEG has 16 channels, so the 16 × 6 feature matrix is then one of the inputs to the network model. For the adjacency matrix A representing the brain functional network, most studies define it by the spatial distance between nodes or certain calculation methods, which only results in direct connections when the spatial distance is very close. This can result in loss of information on long-distance connections. In addition, most GCN now binarize all the connection relations when constructing the adjacency matrix, which seriously ignores the influence of important edges. To solve the above problems, this study uses the Spearman’s rank correlation coefficient to obtain the continuity value to measure the connection relationship among nodes rather than the spatial distance or binarization value. In conclusion, the Spearman’s rank correlation coefficient (SCC) matrix between relatively pure EEG signals needs to be calculated first. When the value of Spearman’s rank correlation coefficient is between [−1,1], a negative value will appear. In order to calculate the degree matrix D, the criterion of the analysis graph must not consider the polarity of the correlation, that is, whether the correlation is positive or negative. Therefore, the Spearman’s rank correlation coefficient absolute value matrix |SCC| is introduced. In this paper, the adjacency matrix A representing the brain functional network is redefined by the equation (8).


(8) A= |SCC|−I

A is 16 by 16, and I is the identity matrix. In this case, the ith diagonal element of the degree matrix D can be calculated by equation (9).


(9) $$D_{ii}=\overset{16}{\underset{j=1}{\sum }}A_{ij}$$

The input matrices A and H are now obtained, but if the two matrices are multiplied directly at this point, the original distribution of the features will be changed. In the case of a multilayer network, the features will be increasingly different from the input features after several layers of changes, creating some unpredictable problems. Therefore, the adjacency matrix A is symmetrically normalized so that the sum of each row and column is 1. The equation (10) is shown as follows.


(10) $$\tilde{A}=D^{-\frac{1}{2}}AD^{-\frac{1}{2}}$$

**Figure 8:**
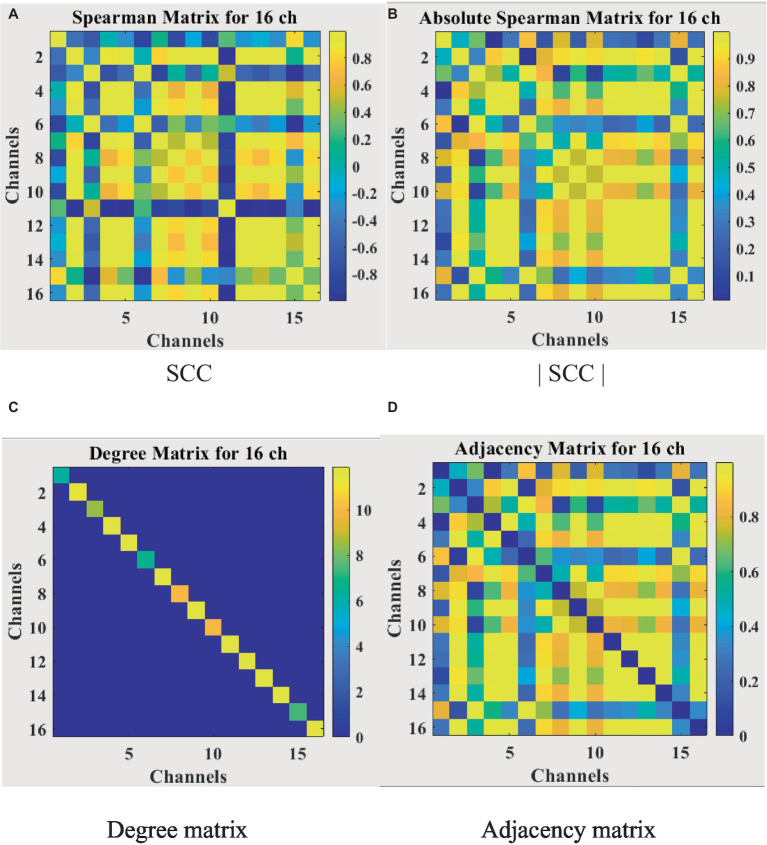
Correlation matrix visualization. **(A)** SCC. **(B)** |SCC|. **(C)** Degree matrix. **(D)** Adjacency matrix.

$\tilde{A}$ is the final adjacency matrix. The whole calculation process is visualized in Figure 8, where Figure 8A shows the Spearman’s rank correlation coefficient matrix obtained in the first step, Figure 8B shows the Spearman’s rank correlation coefficient absolute value matrix, Figure 8C shows the degree matrix which is a diagonal matrix, and Figure 8D shows the final adjacency matrix whose main diagonal is 0 and symmetric about the main diagonal. The adjacency matrix contains the spatial information of EEG, unlike traditional neural network inputs that only have temporal information, which will improve the accuracy of depression recognition.

#### 2.4.4 W-GCN-GRU

**Figure 9:**
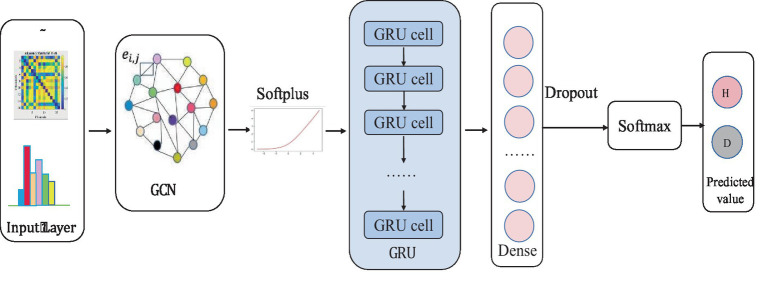
W-GCN-GRU network structure.

The input layer to the model is a symmetric normalized A first left multiplied by H and then left multiplied by the weight matrix W. A nonlinear smoothed rectified linear unit (Softplus) activation function is applied to the graph convolution layer, which prevents gradient vanishing. The GRU acts as a secondary network to the GCN. This is followed by a dense layer that unites all relevant features assigned weights. To reduce the risk of overfitting, this study uses a dropout layer with a probability of 0.2 between the dense layer and the output layer. The final layer is a classifier function, Softmax, which maps the output values of the neurons in the previous layer to the (0, 1) interval. This probability value, which is greater than 0 and less than 1, is the probability of a category, enabling binary classification for depression recognition. The specific network parameters were determined by the grid search method. Ultimately, the detailed design of the W-GCN-GRU network is shown in Figure 9, and the specific structural hierarchy is shown in Table 6.

**Table 6:** W-GCN-GRU network layers.

Layer	Structure
1st	Input layer ( $\tilde{A}$ :16*16, H:16*6, W:6*6)
2nd	GCN (H:16*6, W:6*6)
3rd	GRU
4th	Dense (dropout: 0.2)
5th	Softmax

Some of the other technical details included in the W-GCN-GRU network model are listed below:

1. Activation function

In this study, we use the nonlinear smooth rectified linear unit (Softplus) as the activation function in our proposed method. Softplus has the advantage of more efficient gradient descent and backpropagation than other activation functions and avoids the gradient explosion and gradient disappearance problems to a certain extent. The expression for Softplus is given in equation (11).


(11) $$Softplus\left(x\right)=\log\left(1+e^{x}\right)$$

1. Loss function

When using neural networks for classification tasks, a loss function is generally required to evaluate the model performance and measure the classification accuracy. In this study, we use the binary_crossentropy loss function as the loss function for hybrid networks, which is also a loss function often used in binary classification tasks. The binary cross entropy function is given in equation (12).


(12) $$loss=-\frac{1}{N}\overset{N}{\underset{i=1}{\sum }}y_{i}*\log\left(\tilde{y_{i}}\right)+\left(1-y_{i}\right)*\log\left(1-\tilde{y_{i}}\right)$$

where N denotes the number of samples, $y_{i}$ denotes the true label 0 or 1, and $\tilde{y_{i}}$ denotes the probability of the label predicted by the model.

1. Optimizer

In order to find the most optimal parameter that makes the value of the loss function as small as possible, this paper conducts a comparative experiment on the MODMA dataset for depression recognition. The accuracy and loss rates of the four optimizers were compared for different epochs, and the results are shown in Tables 7, 8, where it can be seen that the Adam optimizer performed the best.

**Table 7:** Classification accuracy of different optimizers under different epochs (Accuracy).

**Optimizer**	**Epoch=20**		**Epoch=50**		**Epoch=100**	
	Training set accuracy	Test set accuracy	Training set accuracy	Test set accuracy	Training set accuracy	Test set accuracy
SGD	76.2%	74.6%	81.46%	80.02%	87.37%	86.21%
Momentum	81.8%	80.1%	86.24%	84.78%	90.21%	89.29%
RMSProp	85.9%	84.6%	89.5%	89.99%	91.67%	91.14%
**Adam**	**87.8%**	**86.5%**	**91.3%**	**90.88%**	**92.680%**	**92.30%**

**Table 8:** Cross entropy loss of different optimizers under different epochs (Loss).

**Optimizer**	**Epoch=20**		**Epoch=50**		**Epoch=100**	
	Training set loss	Test set loss	Training set loss	Test set loss	Training set loss	Test set loss
SGD	42.6%	49.8%	40.92%	47.76%	38.30%	42.45%
Momentum	33.8%	42.9%	27.34%	37.72%	25.21%	32.72%
RMSProp	30.3%	34.1%	24.90%	27.44%	23.75%	24.23%
**Adam**	**26.7%**	**28.5%**	**19.42%**	**21.35%**	**18.52%**	**20.20%**

Adam (Kingma and Ba, 2014) was ultimately chosen as the optimizer for the model in this study, with its learning rate lr set to 0.001, primary momentum coefficient $\beta_{1}$ set to 0.9, and secondary momentum coefficient $\beta_{2}\;\;$ set to 0.999.

### 2.5 W-GCN-GRU network parameters

In this study, the scikit-learn-based grid search method (Cong et al., 2021) was chosen to tune the network parameters and hyperparameters to determine the best combination of parameters for the model. The grid search method has higher efficiency and faster efficiency than the random search and Bayesian optimization methods.

1. Network parameter tuning

**Figure 10:**
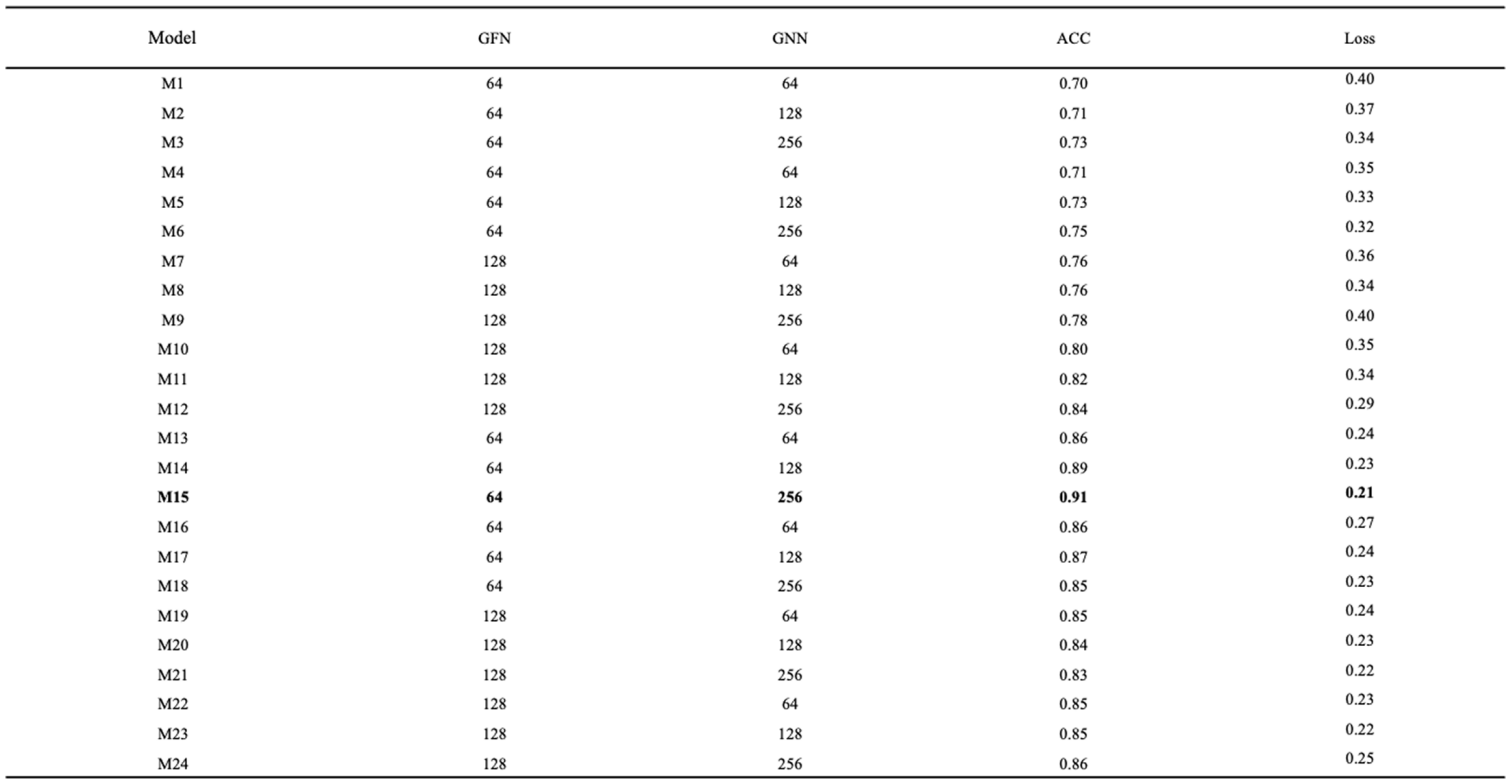
Optimization of network parameters.

When tuning the network parameters, it is sufficient to select a portion of the data. In this study, one-tenth of the data volume, i.e., 1,590 samples, were randomly selected from the MODMA dataset for the experiment. Firstly, epoch and batch size were set to 10 and 128, respectively. Then the number of graph filters in the graph convolution layer and the number of neurons in the GRU were defined as GFN and GNN, respectively, for tuning. The grid search results are shown in Figure 10, and it can be seen that the model numbered M15 has the best performance with an accuracy rate of 0.91 and a loss rate of 0.21.

1. Hyperparameter tuning

**Figure 11:**
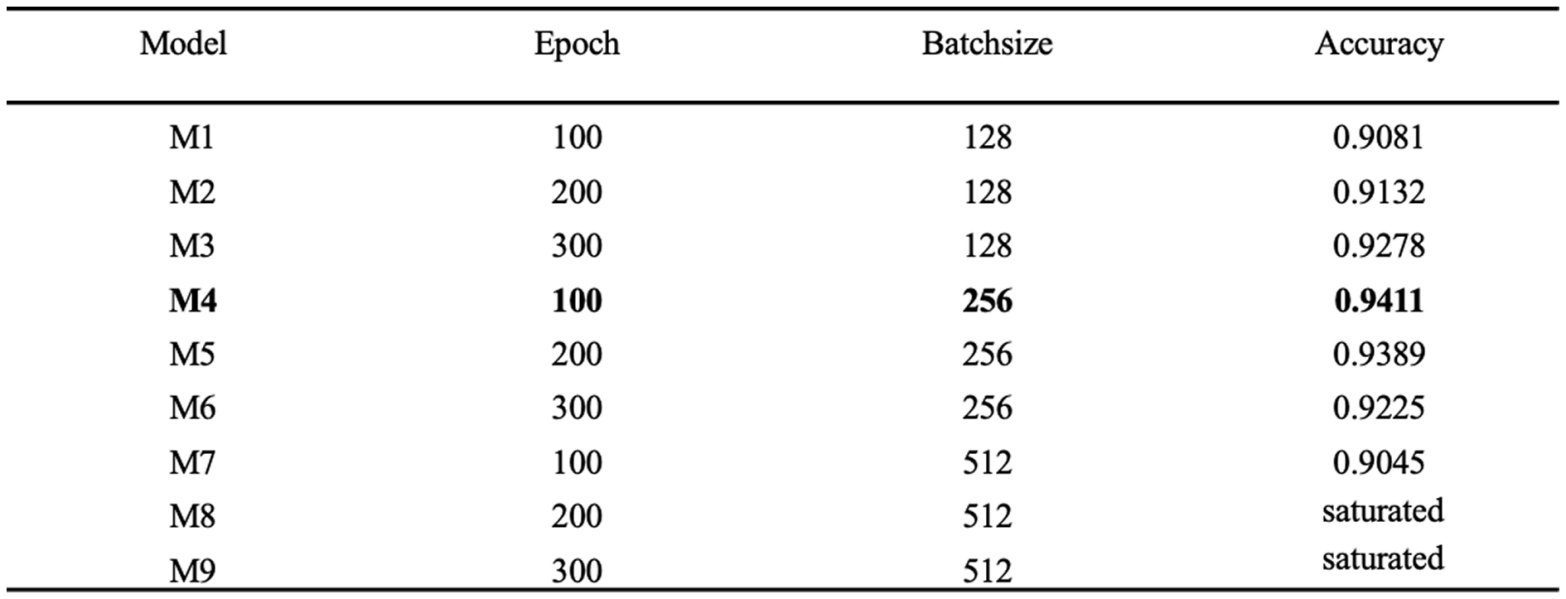
Hyperparameter tuning.

Hyperparameter tuning is the process of determining the correct combination of hyperparameters to maximize the performance of a model. Currently the main hyperparameter tuning methods are random search, grid search and Bayesian optimization. In the random search method, random combinations of hyperparameters are tried from the network at each iteration, the performance is recorded and finally the best performing combination of hyperparameters is obtained. In the grid search method, a grid of possible values is created for the hyperparameters, combinations of hyperparameters are tried in a particular order at each iteration and the model performance is recorded and finally, the best model with the best hyperparameters is returned. Bayesian optimization finds the smallest point in the least number of steps, and it uses an Acquisition Function (Acquisition Funtion) that directs the sampling to the region that has the potential to be better than the current best observation. Among them, the grid search method has higher efficiency and faster efficiency. scikit-learn is commonly used tool for grid search. In this study, scikit-learn based grid search method is chosen to tune the network parameters and hyperparameters and find the best combination of parameters for the model. Epoch and batch size are very important hyperparameters in neural network models. In this paper, epoch and batch size are tuned by the grid search method based on the optimal combination obtained by tuning the network parameters. As shown in Figure 11, the model performs best when epoch and batch size are 100 and 256, respectively, achieving an accuracy of 0.9411.

1. Other core hyperparameters

Other related core hyperparameters are set in Table 9 according to common conclusions. The learning rate was 0.001. It represents the step size by which the gradient moves towards the optimal solution of the loss function in each iteration, and its size determines how fast the network learns. A suitable learning rate allows the loss function to converge to a minimum at a suitable rate. Decay rate and learning rate are inextricably linked. Typically, a larger learning rate will cause the model to learn quickly but may skip the optimal solution, while a smaller learning rate may slow down the learning process but allow for a more accurate finding of the optimal solution. Decay rate is a strategy that adjusts the learning rate, decreasing it as training progresses, to help the model find the optimal solution more accurately. Decay steps is a metric that decays every how many rounds of iteration, and can be called the decay rate. In this paper, the decay rate and decay steps are 0.98 and Total sample/Batchsize, respectively, which represent that the learning rate is multiplied by 0.98 once for every Total sample/Batchsize iteration.

**Table 9:** Else hyperparameter.

Hyperparameter	Value
Learning rate	0.001
Decay rate	0.98
Decay steps	Total sample/batch size

## 3 Results

### 3.1 The process of identifying depressive states

**Figure 12:**
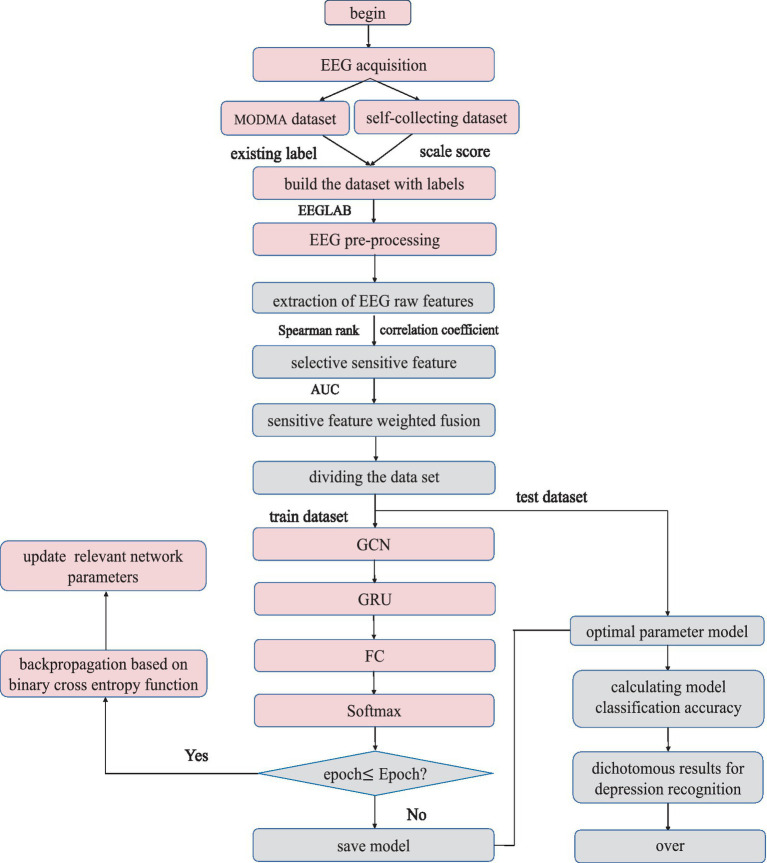
W-GCN-GRU algorithm flow.

Based on the parameter-tuned network model for depressive state identification, the specific algorithm flow is shown in Figure 12.

The specific steps are as follows:

Step 1: For self-collected EEG, the dataset is labeled according to the scale score. The MODMA dataset has been divided into depression and health, i.e., labels already exist.
Step 2: The raw EEG data was preprocessed to remove various artefacts with the help of MATLAB and EEGLAB.
Step 3: A total of 23 dimensional raw features in the time domain, frequency domain, and nonlinearity are extracted from the relatively pure EEG signal.
Step 4: The Spearman’s rank correlation coefficient was introduced for feature dimension reduction to obtain 6-dimensional sensitive features.
Step 5: The AUC assigns different weight coefficients to each sensitive feature to complete the weighted fusion of features.
Step 6: Divide the dataset into a training set and a test set. The training set trains the W-GCN-GRU network model, the verification set updates the weights and biases in reverse, and saves the trained model.
Step 7: The test set verifies the effectiveness and sensitivity of the algorithm. The performance of the depression state recognition model was evaluated based on predictions and real labels.

### 3.2 Evaluation metrics

In the classification task, we select the following index to evaluate the performance of the model.

1. Accuracy is defined as the ratio of the number of correctly classified samples to the total sample for a given test dataset. Its formula is given in equation (13).

(13) $$Accuracy=\frac{\left|TP\right|+|TN|}{\left|TP\right|+\left|FP\right|+\left|TN\right|+|FN|}$$

However, in the case of unbalanced positive and negative samples, this indicator has major flaws. For example, there are 1,000 samples with 900 positive samples and only 100 negative samples. Even if the classification model predicts all samples as positive, the accuracy is 90%. Clearly, this metric is not convincing, so other metrics must be used to evaluate the model’s performance in combination.

1. Precision, which is the ratio of the number of correctly classified positive samples to the number of classified positive samples, measures the accuracy of the check. Its formula is given in equation (14).

(14) $$Precision=\frac{\left|TP\right|}{\left|TP\right|+\left|FP\right|}$$

1. Recall, which is the ratio of the number of correctly classified positive samples to the number of actual positive samples, measured as the rate of complete. Its formula is given in equation (15).

(15) $$Recall=\frac{\left|TP\right|}{\left|TP\right|+|FN|}$$

1. F1 score is to evaluate the pros and cons of different algorithms. The concept of F1 value is proposed based on Precision and Recall. Its formula is given in equation (16).

(16) $$F1-\mathrm{score}=\frac{2*\mathrm{Precision}*\mathrm{Recall}}{\mathrm{Precision}+\mathrm{Recall}}$$

where TP, FP, TN, and FN represent true positive, false positive, true negative, and false negative, respectively. Taking depression recognition as an example, true positive represents the number of samples predicted to be depressed and actually depressed; false positive represents the number of samples predicted to be depressed and actually healthy; true negative represents the number of samples predicted to be healthy and actually healthy; and false negative represents the number of samples predicted to be healthy and actually depressed.

1. Confusion matrix

Confusion matrix is also an effective model evaluation index, which can more intuitively show the classification accuracy of the dataset. The horizontal axis is the predicted value, and the vertical axis is the true value.

### 3.3 Results and analysis

Experiments were conducted on the public dataset and the self-collected dataset, respectively, to verify the efficiency of the proposed hybrid network. H and D represent the healthy and depressed populations, respectively, and the distribution of samples in the two datasets is shown in Table 10.

**Table 10:** Sample distribution in dataset.

Dataset	H	D	Total	Subjects (H/D)
MODMA dataset	8,700	7,200	15,900	29/24
Self-collected dataset	4,500	3,600	8,100	25/20

#### 3.3.1 Classification results on MODMA

**Figure 13:**
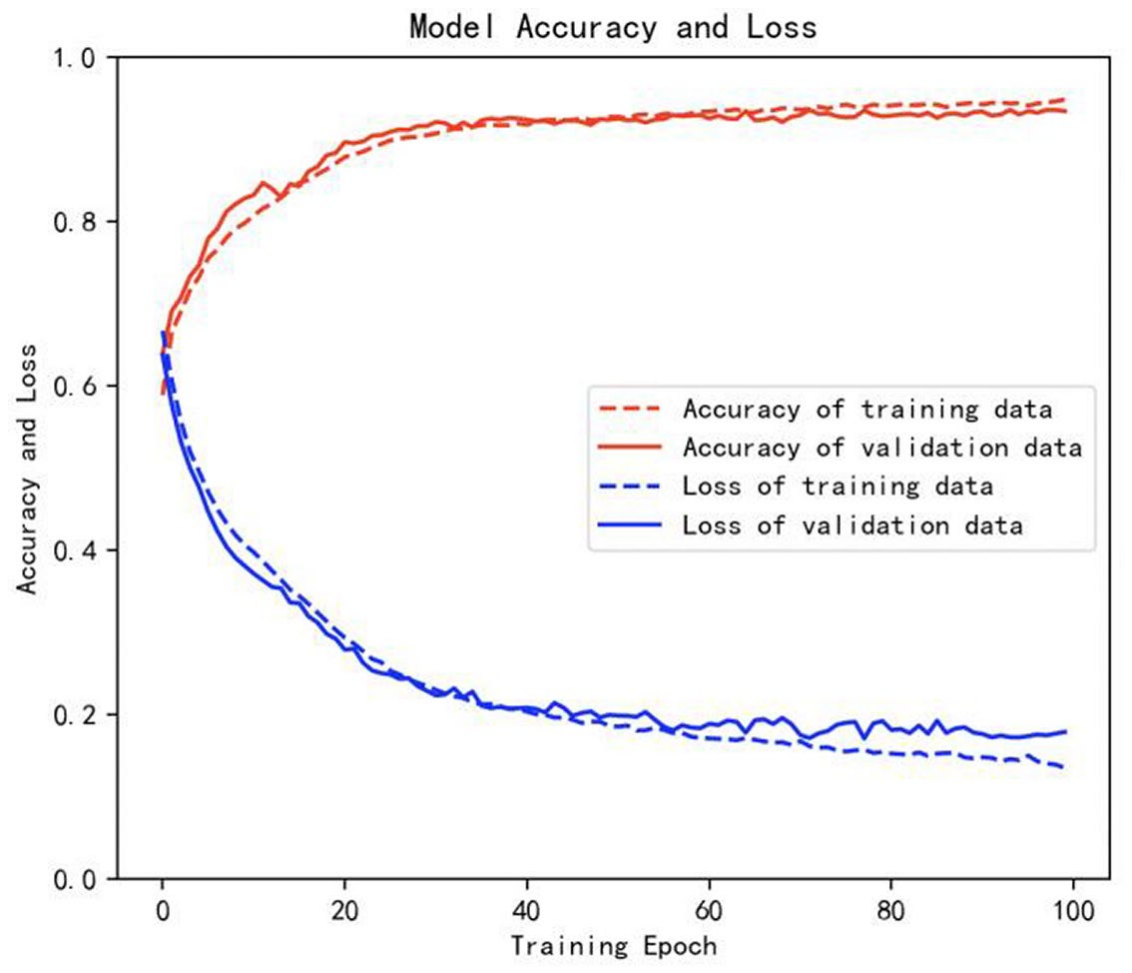
Iterative curve of training process.

**Figure 14:**
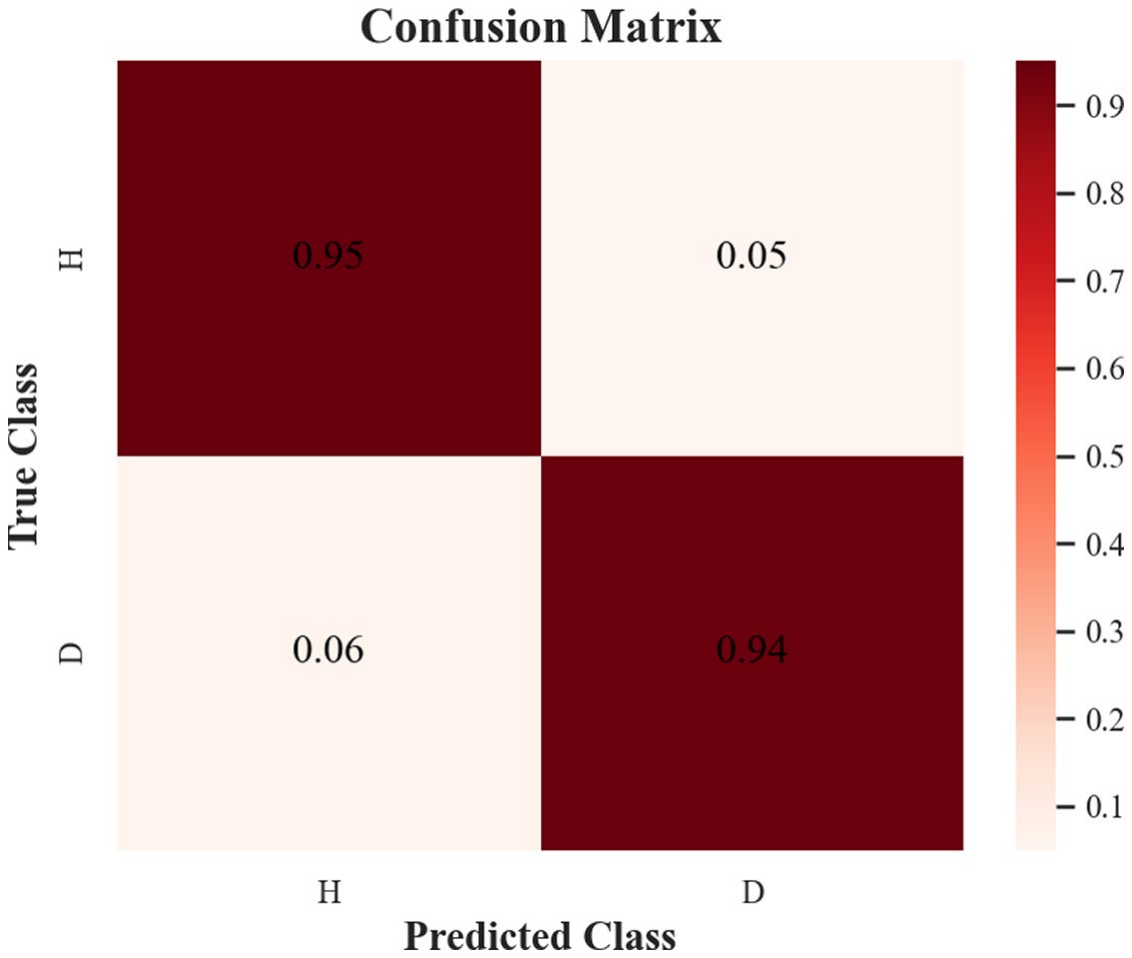
Confusion matrix of the test set.

The samples in the public dataset are divided into a training set and a test set in the ratio of 9:1, and then one-tenth of the training set is selected as the validation set. Figure 13 shows the iteration curves of the training process of the W-GCN-GRU model. The red dashed line indicates the accuracy of the training data, the red solid line indicates the accuracy of the validation data, the blue dashed line indicates the loss rate of the training data, and the blue solid line indicates the loss rate of the validation data. From the experimental results, we know that the accuracy of the training set is 94.72%, the loss rate is 15.48%, the accuracy of the validation set is 93.68%, and the loss rate is 17.96%. The training processes all performed well on the models, converged quickly, and no overfitting occurred. This demonstrates that the method proposed in this study is not only effective for depression recognition, but also has high classification accuracy. Figure 14 shows the confusion matrix classification results of the W-GCN-GRU model on the test set. The calculation shows that the classification accuracy on the test set is 94.5%.

Table 11 lists the classification reports of depression status identification on the W-GCN-GRU model in the public dataset. The precision, recall, and F1 values are all above 0.94, which proves that the method proposed in this study can effectively identify depression.

**Table 11:** Classification report.

Description	Label	Precision	Recall	F1
Depressive state	0	0.95	0.94	0.94
Health	1	0.94	0.95	0.94

To verify the improvement of model speed by feature dimension reduction and the improvement of model average accuracy by sensitive feature weighting, this study conducted comparative experiments based on the W-GCN-GRU network. The experimental results are shown in Tables 12, 13. From the two tables, it can be seen that feature dimensionality reduction improved the model speed by 75% and feature weighting fusion improved the average accuracy of the model by 2.2%, both of which improved the performance of the model.

**Table 12:** Feature dimension reduction comparison.

	No feature dimension reduction	Performing feature dimension reduction
Running time/s	920	234
Speed boost	0%	75%

**Table 13:** Feature weighted fusion comparison.

	No feature weighting fusion	Perform feature weighted fusion
Average accuracy	90%	92%
Average accuracy improvement	0%	2.2%

**Figure 15:**
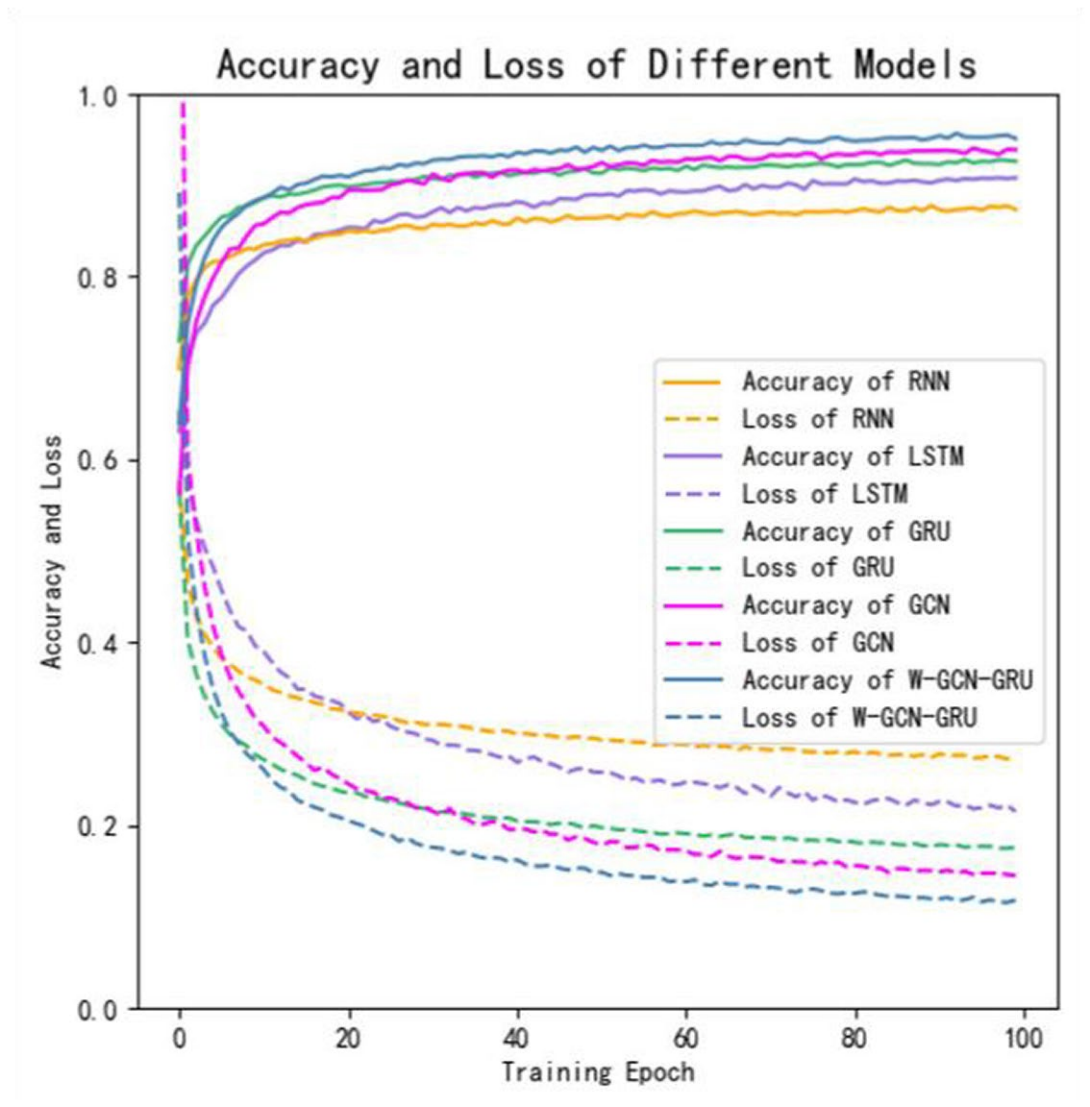
Comparative experimental results on MODMA dataset.

**Figure 16:**
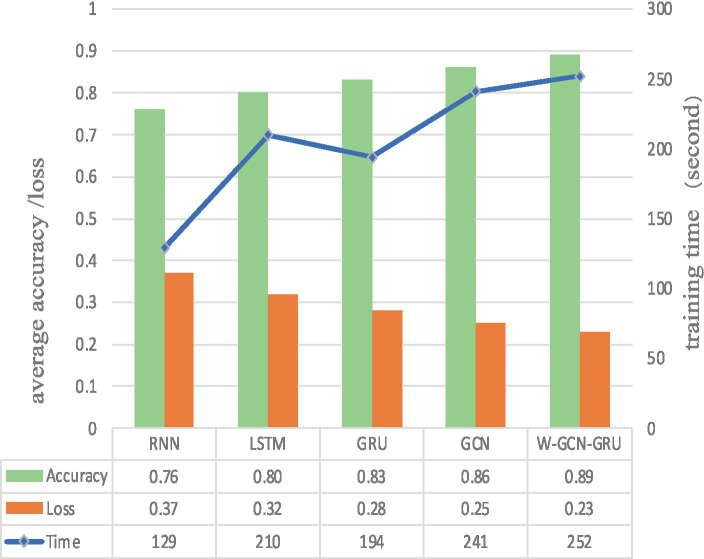
Average accuracy, loss, and training time of different network models.

To further evaluate the performance of the proposed method, four sets of cross-sectional comparison experiments were conducted in this study, i.e., W-GCN-GRU was compared with RNN, LSTM, GRU, and GCN networks on the MODMA dataset. Figure 15 shows the training curves for the five models, where the solid line represents accuracy and the dashed line represents loss. The iterative curves show that the GCN-fused GRU network of this study outperforms the other neural networks in recognition, with the highest accuracy rate for depression recognition and the smallest value of the loss function. In order to verify the high performance of W-GCN-GRU model from multiple aspects, Figure 16 compares the average accuracy, average loss, and training time of these five deep learning algorithms during training. As can be seen in Figure 16, the W-GCN-GRU model has the highest average accuracy of 89% and the lowest average loss rate of 23%. Although the average training time of this model is slightly longer, the performance is still optimal when taken together. The reason for the long average training time may be related to the complex structure of the model.

#### 3.3.2 Classification results on self-collecting dataset

**Figure 17:**
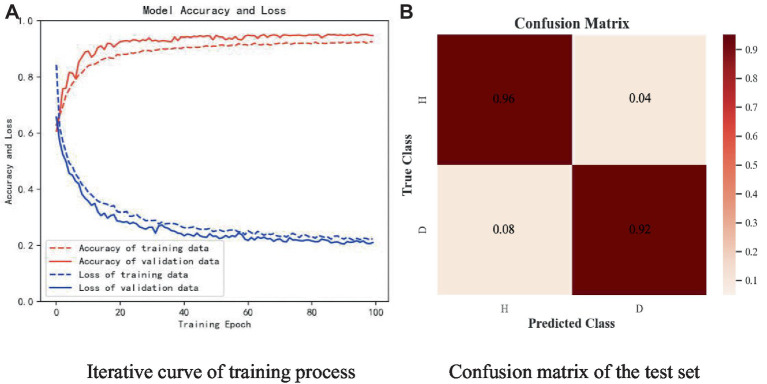
Experimental results of self-collected dataset. **(A)** Iterative curve of training process. **(B)** Confusion matrix of the test set.

The subjects of the experiment were all in the age group of 17-28. there were 25 healthy controls with a male to female ratio of 13:12 and 20 patients with depressive states with a male to female ratio of 14:6. The sampling frequency of the experiment was 250 Hz. Like the open dataset, the real dataset is divided into the training set and the test set in a 9:1 ratio, and one-tenth of the training set is used as the verification set. Figure 17 shows the experimental results of W-GCN-GRU model on the self-collecting dataset. In diagram a, the red dashed line represents the accuracy of training data 91.58%, the red solid line represents the accuracy of verification data 93.76%, the blue dashed line represents the loss rate of training data 22.34%, and the blue solid line represents the loss rate of verification data 21.02%. Diagram b shows the confusion matrix of the test set, which further indicates that the model performs well and has good generalization ability.

**Figure 18:**
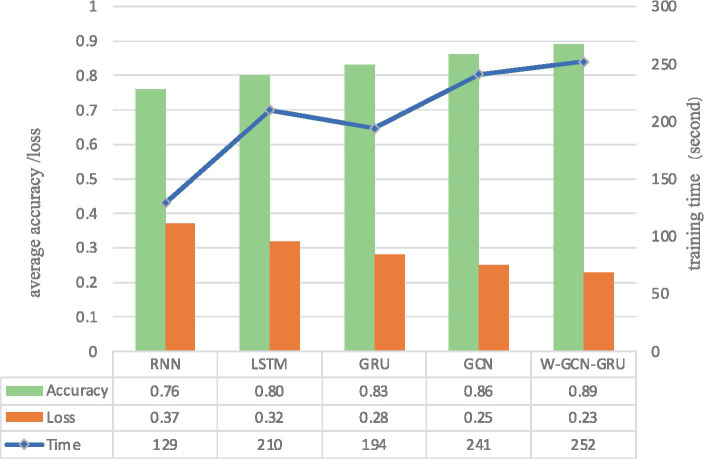
Average accuracy, loss, and training time of different network models.

Figure 18 compares the average accuracy, loss, and time of the proposed method in this study with RNN, LSTM, GRU, and GCN, and it can be seen that the W-GCN-GRU model proposed in this study has the highest accuracy and the lowest loss. Although it takes a little longer, overall, it has the highest performance.

**Figure 19:**
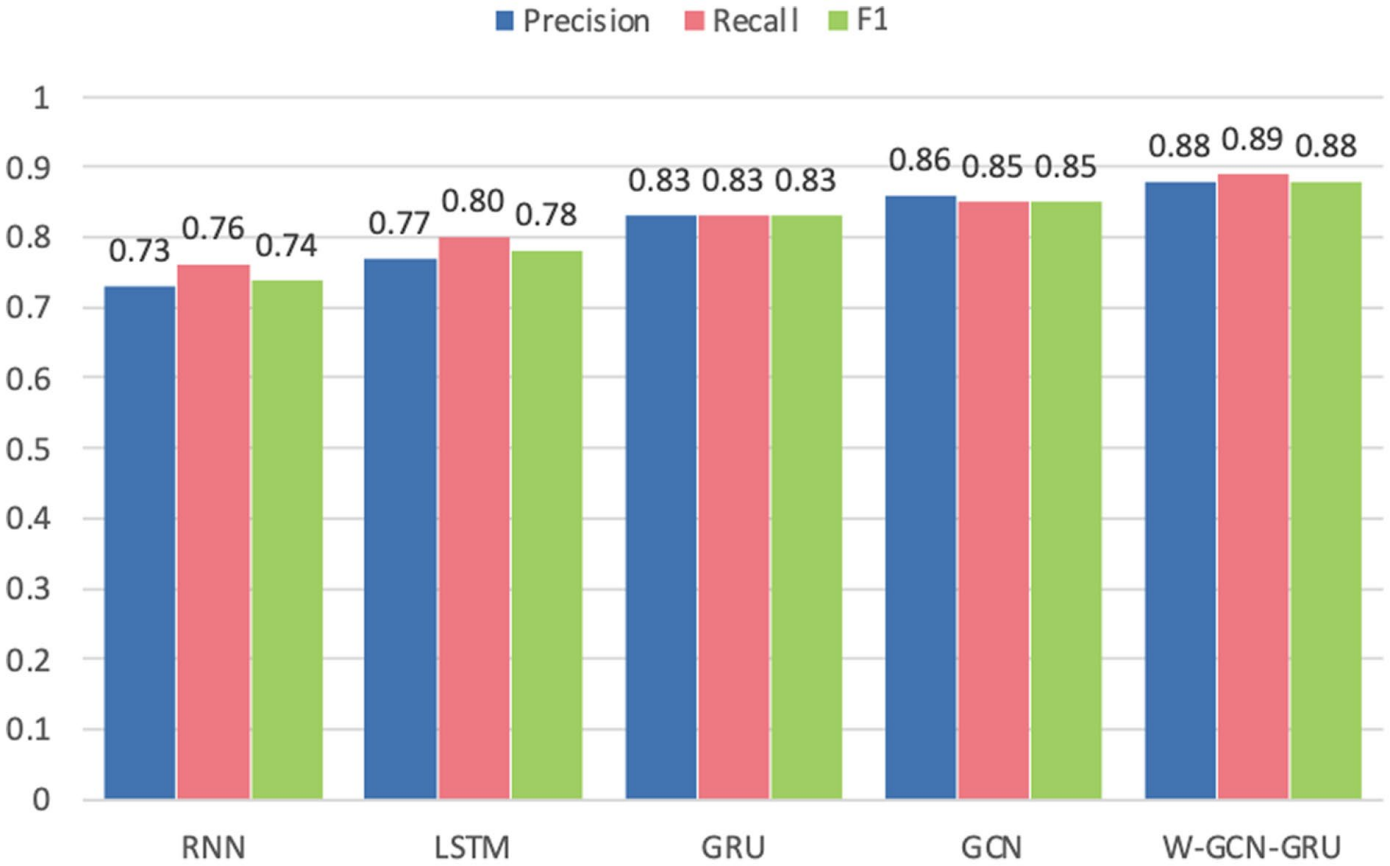
Comparison of evaluation indexes of each model.

The average precision, recall, and F1 values of each model are shown in Figure 19.

Through the experimental evaluation of effectiveness and sensitivity on the two types of datasets, there is no doubt that W-GCN-GRU neural network has the most efficient depression recognition performance in health and depression, and the classification accuracy is up to 94.72%.

## 4 Discussion

For comparison, we select 6 existing model in the field of depression state recognition. These methods mainly use nonlinear features and brain functional network. Table 14 demonstrates the specific comparisons. (Sun et al. 2020; Zhang Lu, 2022) used the same public dataset in their paper. Sun used phase lag index (PLI) to study the brain functional connectivity network, selected four different types of brain electrical features including PLI, and used multiple classifiers to identify depression, reaching the highest accuracy of 83.5%. Zhang Lu, (2022) selected a total of 54 features. Compared with different feature selection algorithms and experimental results of classifiers, the classifier based on Soft Voting algorithm has the best result. Rong, (2020) investigated a functional connectivity matrix-based convolutional neural network model for mild depression recognition. Different graph-theoretic approaches were used to construct the functional brain network and the classification results showed that the coherence approach had the best recognition performance, obtaining an accuracy of 85.62%.For our study, we conducted experiments on the public dataset MODMA and self-collected dataset with the aim of exploring methods and models that can recognize depressive states with high performance. We investigated sensitive feature selection and weighted fusion methods, while obtaining the adjacency matrix representing the functional brain network thus constructing the depression state recognition model W-GCN-GRU. The study has the following key observations.Firstly, based on the original 23-dimensional feature set extracted from time, frequency and nonlinear domains, this study proposes a high-quality feature downscaling method. The method combines Spearman’s rank correlation coefficient and thresholding to perform feature dimensionality reduction on the original 23-dimensional features, which yields six sensitive features, removes redundant features, reduces algorithmic complexity, and improves the experimental speed by 75% after feature dimensionality reduction. Secondly, in order to focus the network on more representative features, this paper uses AUC to assign different weight factors to sensitive features. Iterating over the sensitive features matrix yields weight coefficients for the six sensitive features, which are normalized to be 0.08, 0.31, 0.12, 0.22, 0.15, and 0.12, respectively. Ultimately, the accuracy of the network model using weighted features was improved by 2.2%.Thirdly, the continuity values measuring the functional connectivity between nodes are obtained based on the Spearman rank correlation coefficient, based on which an adjacency matrix representing the functional brain network was obtained. Adjacency matrix mines the topological structure information of brain regions, fully extracts the spatial features between EEG data, which improves the accuracy of depression recognition from a spatial perspective. At last, the depression recognition model W-GCN-GRU fully considers the spatiotemporal information interaction of EEG and achieves 94.72% accuracy, which is better and more efficient than other deep learning model.Although this paper has carried out innovative research in feature processing and network model construction to improve the accuracy of depression state recognition, there is still problems to be further studied and explored as follows. Firstly, for GCN networks, increasing the number of electrodes in the electrode cap will result in richer spatial information, and it is unknown whether it will improve the accuracy of depression recognition. Future research could start by increasing the number of electrode channels. Secondly, when constructing the feature matrix of the nodes, the same 6-dimensional features are all chosen. However, the spatial locations of the nodes are different and the feature information may be different. Therefore, the next study can start from each node to get the most representative feature information. Zong et al. (2023) used the random search method to select the discriminative features for each channel. Maybe we can receive inspiration from it. Thirdly, it is expected that attempts will be made to synchronize the acquisition of multiple physiological signals from the subjects and to take advantage of the complementarity between multimodal data to improve depression recognition accuracy.

**Table 14:** Comparison of accuracy and loss of different networks.

Author	Year	Normal/depressed	Features	Classifier	Accuracy
Hajian et al. (2019)	2018	13/13	HFD, KFD	Logistic regression	92%
Mahato and Paul (2019)	2019	30/34	Linearity and nonlinearity	Machine learning	91.67%
Rong (2020)	2020	24/24	PSD	CNN	85.62%
Sun et al. (2020)	2020	**29/24**	L + NL + PLI + NM	C4.5, BFD, LR and so on	83.5%
Chen (2020)	2020	28/27	Microstates, brain networks	SVM	90%
Zhang Lu (2022)	2022	**29/24**	54 features	Soft voting	82.16%
**This paper**	2023	**29/24**	$E\;\left(\delta \right),\;E_{\mathrm{all}}$ , Kurtosis, Skewness, HjorthMob, and DFAs	W-GCN-GRU	94.72%

The bold values is the subject number of Normal / depression.

## 5 Conclusion

This article examines key issues in high-performance recognition of depressive states based on EEG. It makes sense to perform feature downscaling and sensitive feature-weighted fusion, improving the training speed and accuracy by 75% and 2.2%, respectively. In addition, we creatively propose a W-GCN-GRU depressive state recognition neural network based on weighted sensitive features. In particular, an adjacency matrix representing the functional brain network was constructed based on the Spearman rank correlation coefficient as one of the inputs to the GCN network. Compared to RNN, LSTM, GRU, and GCN networks, Our proposed method has the highest recognition performance, achieving 94.72% accuracy and 15.48% loss rate.

## Data availability statement

The raw data supporting the conclusions of this article will be made available by the authors, without undue reservation.

## Ethics statement

The studies involving humans were approved by the Ethics Committee of Science and Technology, Beijing University of Technology. The studies were conducted in accordance with the local legislation and institutional requirements. The participants provided their written informed consent to participate in this study. Written informed consent was obtained from the individual(s) for the publication of any potentially identifiable images or data included in this article.

## Author contributions

ZW: Writing – review & editing, Project administration. CH: Writing – review & editing, Methodology, Validation, Writing – original draft. WL: Writing – review & editing, Investigation. XiaZ: Writing – original draft, Software, Visualization. XixZ: Writing – review & editing, Data curation.
